# CDDO regulates central and peripheral sensitization to attenuate post‐herpetic neuralgia by targeting TRPV1/PKC‐δ/p‐Akt signals

**DOI:** 10.1111/jcmm.18131

**Published:** 2024-03-01

**Authors:** Chun‐Ching Lu, Chia‐Yang Lin, Ying‐Yi Lu, Hung‐Pei Tsai, Chih‐Lung Lin, Chieh‐Hsin Wu

**Affiliations:** ^1^ Department of Orthopaedics and Traumatology National Yang Ming Chiao Tung University Hospital Yilan Taiwan; ^2^ Department of Orthopaedics, School of Medicine National Yang Ming Chiao Tung University Taipei Taiwan; ^3^ Department of Orthopaedics and Traumatology Taipei Veterans General Hospital Taipei Taiwan; ^4^ Department of Nuclear Medicine Kaohsiung Medical University Hospital Kaohsiung Taiwan; ^5^ Department of Dermatology Kaohsiung Veterans General Hospital Kaohsiung Taiwan; ^6^ Department of Post‐Baccalaureate Medicine, School of Medicine, College of Medicine National Sun Yat‐sen University Kaohsiung Taiwan; ^7^ Shu‐Zen Junior College of Medicine and Management Kaohsiung Taiwan; ^8^ Division of Neurosurgery, Department of Surgery Kaohsiung Medical University Hospital Kaohsiung Taiwan; ^9^ Department of Surgery, School of Medicine, College of Medicine Kaohsiung Medical University Kaohsiung Taiwan; ^10^ Center for Big Data Research Kaohsiung Medical University Kaohsiung Taiwan; ^11^ Drug Development and Value Creation Research Center Kaohsiung Medical University Kaohsiung Taiwan

**Keywords:** analgesic, neuroprotective, postherpetic neuralgia (PHN), sensitization

## Abstract

Postherpetic neuralgia (PHN) is a notorious neuropathic pain featuring persistent profound mechanical hyperalgesia with significant negative impact on patients' life quality. CDDO can regulate inflammatory response and programmed cell death. Its derivative also protects neurons from damages by modulating microglia activities. As a consequence of central and peripheral sensitization, applying neural blocks may benefit to minimize the risk of PHN. This study aimed to explore whether CDDO could generate analgesic action in a PHN‐rats' model. The behavioural test was determined by calibrated forceps testing. The number of apoptotic neurons and degree of glial cell reaction were assessed by immunofluorescence assay. Activation of PKC‐δ and the phosphorylation of Akt were measured by western blots. CDDO improved PHN by decreasing TRPV1‐positive nociceptive neurons, the apoptotic neurons, and reversed glial cell reaction in adult rats. It also suppressed the enhanced PKC‐δ and p‐Akt signalling in the sciatic nerve, dorsal root ganglia (DRG) and spinal dorsal horn. Our research is the promising report demonstrating the analgesic and neuroprotective action of CDDO in a PHN‐rat's model by regulating central and peripheral sensitization targeting TRPV1, PKC‐δ and p‐Akt. It also is the first study to elucidate the role of oligodendrocyte in PHN.

## 1 INTRODUCTION

Dysfunction of central or peripheral nervous system following nerve injury due to acute events or systemic disease induces neuropathic pain.^1^ Postherpetic neuralgia (PHN) is a notorious neuropathic pain that can last for months to years, which features persistent profound mechanical allodynia and spontaneous pain.^2^ Around 10%–35% of patients who recover from herpes zoster virus infection suffer from this severe complication.^3^ It typically occurs in elderly and results in depression, sleep disturbances, and social withdrawal. Both allodynia and spontaneous pain often coexist.^4^ The intensity of mechanical allodynia increases with the intensity of spontaneous pain, which is positively correlated to the severity of anxiety and depression. As an intractable chronic pain, PHN often exhibits significant negative impact on patients' daily activity and life quality.^5^

From damaged afferent nociceptive fibres to central nervous system (CNS), PHN happens as a consequence of central and peripheral sensitization triggered by neurogenic inflammations.^6^ Reactivation from dorsal root ganglia, virus elicits damage to sensory nerves to induce burning, stinging, allodynia, and hyperalgesia in PHN.^7^ Compared with herpes zoster patients without neuralgia, diminished epidermal fibre, loss of axon in dorsal root ganglia and atrophy of spinal dorsal horns are identified in PHN patients' postmortem studies.^8^ Patients not only have irritable nociceptors, but also develop fibre deafferentation with reorganization of central neurons connections. Reconnections with CNS on affected skin cause persistent allodynia and hyperalgesia.^7^ During acute phase, applying neural blocks may benefit to minimize the PHN risk by interfering central and peripheral sensitization.^9^ Capsaicin can activate C‐nociceptors and prolong the nociceptive input to imitate the PHN symptoms.^10^ Similarly, RTX can impede thermal sensitivity and arise mechanical allodynia, like the complaints of PHN patients. As a result, RTX is often utilized to stimulate PHN as a non‐viral model.^11^

Triterpenoids, traditional Asian medicine synthesized from chrysanthemum flower, exert anti‐inflammatory, and anti‐carcinogenic activities.^12^ CDDO is a first‐in‐class synthetic triterpenoid, which serves as a potent multifunctional molecule. Depending on Keap1/Nrf2 and nuclear factor‐κB (NF‐κB) pathways, it can regulate the balance of redox reaction, inflammatory response, programmed cell death and cell proliferation.^13^ At low concentrations, it interacts with Keal/Nrf2 to activate the phase 2 cytoprotective pathway, whereas it interacts with IκB kinase (IKK) to regulate cell proliferation and apoptosis at higher concentrations. Through both caspase‐dependent and ‐independent pathways, CDDO can induce cell apoptosis in malignant diseases.^14^ It regulates growth and cellular differentiation by modulating peroxisome proliferator activator receptor‐γ (PPARγ) activity and suppressing NF‐κB activity.^15^ It behaves as an anti‐cancer agent in osteosarcoma, breast cancer,^16^ ovarian, prostate, and colon cancers. Moreover, Tran et al. found that CDDO‐derivative provides a neuroprotective effect on dopaminergic neuron‐like MN9D cells by inhibiting TNFα production and modulating microglia activities.^17^

In this study, we aimed to explore whether CDDO could generate analgesic action in a PHN‐rats' model that was caused by an intraperitoneal (i.p.) treatment of RTX. Furthermore, we investigated underlying mechanisms of PHN for peripheral and central sensitization and focused on the involvement of neurons and glial cell reaction. Our findings indicated that CDDO can modulate the pain threshold by limiting the apoptotic activity of neurons, the glial cell reaction, as well as by negatively regulating the PKC‐δ and phosphorylation of Akt pathways.

## 2 MATERIALS AND METHODS

### 2.1 Animals

**FIGURE 1:**
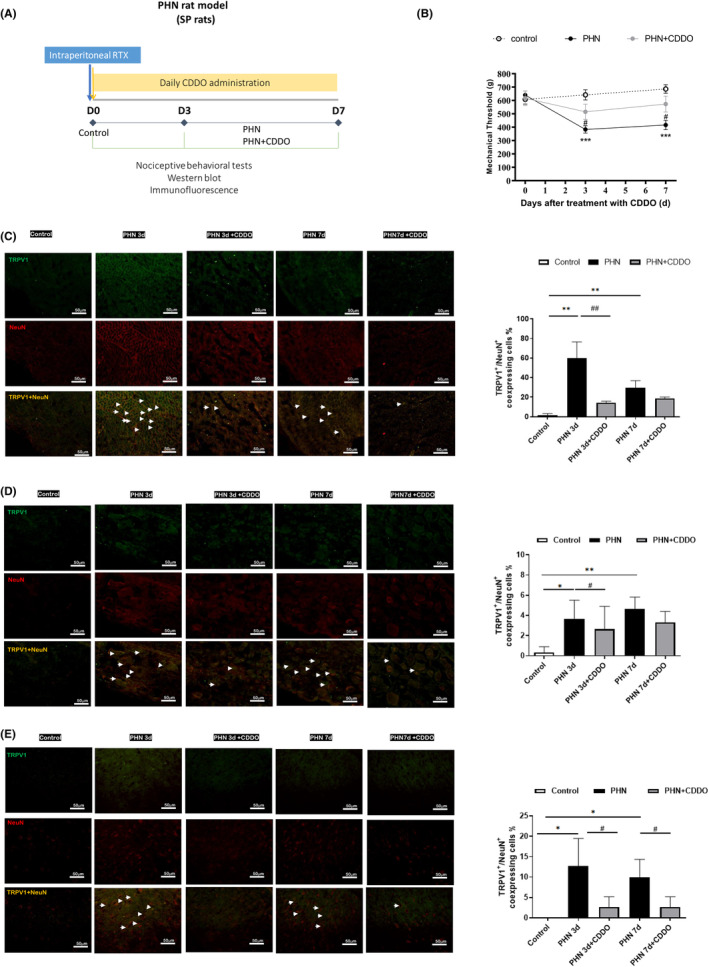
CDDO administration ameliorated PHN. (A) Diagram of the experimental protocol. (B) Nociceptive behavioural testing was conducted at baseline (before injection of RTX) and on the 3rd, and 7th day after the initiation of CDDO treatment. The daily administration of CDDO reverses the mechanical hyperalgesia in PHN‐rats. (*n* = 6/group) (C) The neuronal cell marker, NeuN (red) and TRPV1 staining, representative photograph in sciatic nerve. Quantification of the percentage of TRPV1‐positive neuronal cells over NeuN‐ positive cells. Scale bars = 50 μm. (D) The neuronal cell marker, NeuN (red) and TRPV1 staining, representative photograph in DRG. Quantification of the percentage of TRPV1 staining ‐positive neuronal cells over NeuN‐ positive cells. Scale bars = 50 μm. (E) The neuronal cell marker, NeuN (red) and TRPV1 staining, representative photograph in spinal dorsal horn. Quantification of the percentage of TRPV1 ‐positive neuronal cells over NeuN‐ positive cells. Scale bars = 50 μm. Yellow fluorescence showed colocalization of TRPV1 (blue) with NeuN (red). (*n* = 3/group) The quantitative data shown as the mean ± SEM (*p < 0.05, **p < 0.01, ***p < 0.001, PHN compared with the control group; #*p* < 0.05, ##*p* < 0.01, PHN compared with the indicated PHN + CDDO group).

Experiments were carried out on male adult Sprague–Dawley rats weighting 300–350 g obtained from National Animal Center (Taiwan). Rats were kept individually in cages under controlled temperature of 20°C–24°C and relative humidity 45%–65%, with a 12 h light/dark cycle and food and water allowed ad libitum. Adult rats were randomly allocated into three groups: control, PHN group, and PHN + CDDO group (six per group). Each PHN‐rat was given a single i.p. injection of RTX (50 μg/kg, Sigma, St. Louis, MO).^11^, ^18^, ^19^, ^20^ CDDO was dissolved in the DMSO at the concentration of 50 mg/mL. Each rat in PHN + CDDO group was given intraperitoneally with 10 mg/kg CDDO daily after RTX injection. The rats in the control group received neither RTX injection nor CDDO treatment. The baseline sensitivity of each rat to mechanical stimulation was determined before RTX injection. The experimental protocol was displayed as Figure 1A. All handling procedures were reviewed and approved according to the Institutional Animal Care and Use Committee of Kaohsiung Medical University. The observers were blinded to treatment allocations.

### 2.2 Nociceptive behavioural tests

The behavioural test was evaluated by using calibrated forceps. Brisk withdrawal at the force applied to the paw was considered a result. Tests were performed at baseline before injection of RTX, on the 3rd and 7th after the initiation of CDDO treatment.

The observers were blinded to treatment groups.

### 2.3 Immunofluorescence

Under anaesthesia with Zoletil (50 mg/kg), rats were decapitated. The L4 to L5 segments of the spinal dorsal horn and DRG were quickly dissected and post fixed in 4% paraformaldehyde. On a cryostat, the sections were cut in 8 μm thickness, rinsed in PBS and then incubated at room temperature (RT) in a blocking buffer composed of 0.1% Triton™ X‐100,1% normal goat serum and 0.1% PBS for 1 h. Subsequently, sections were incubated with anti‐NeuN antibody (1:400, MAB377, Millipore), anti‐GFAP antibody (1:400, G3893, sigma), anti‐IBA1 antibody (1:100, 66827‐1‐Ig, proteintech), anti‐phosphor‐p38 antibody (1:100, #9211, Cell Signalling Technology), anti‐NG2 antibody (1:100, 55027‐1‐AP, proteintech), anti‐TRPV1 antibody (1:100, 66983‐1‐lg, proteintech), anti‐cleaved‐caspase 3 antibody (1:100, #9661, Cell Signalling), anti‐PKC‐δ antibody (1:200, 610398, BD), anti‐phospho‐Akt antibody (Ser473) (1:200, #9271, Cell Signalling Technology) for 24 h at 4°C. After washing for several times, sections were incubated with the corresponding secondary antibodies conjugated with Alexa Fluor®: 594‐labelled anti‐mouse IgG (1:500) or 488‐labelled goat anti‐rabbit IgG (1:500) for 1 h. Apoptotic cells were stained by cleaved‐caspase 3. At last, the sections were washed in PBS and cover slipped. Images were captured by a fluorescent microscope (OLYMPUS, Japan). The percentage of GFAP‐positive cells, IBA1‐positive cells, NG2‐positive cells, TRPV1‐positive neurons, cleaved‐caspase 3‐positive neurons, PKC‐δ/GFAP, PKC‐δ/IBA1, PKC‐δ/NG2, PKC‐δ/NeuN, phospho‐Akt/GFAP, phospho‐Akt/IBA1, phospho‐Akt/NG2, phospho‐Akt/NeuN immunoreactive cells and the ratio of neurons surrounded by satellite‐glia cells or activated‐macrophages were counted manually. The intensity of PKC‐δ/GFAP, PKC‐δ/IBA1, PKC‐δ/NG2, PKC‐δ/NeuN, phospho‐Akt/GFAP, phospho‐Akt/IBA1, phospho‐Akt/NG2 and phospho‐Akt/NeuN immunoreactive cells were measured by ImageJ software (NIH, Bethesda, USA).

### 2.4 Western blot

Under anaesthesia with Zoletil (50 mg/kg), rats were decapitated. The L4 to L5 segments of the spinal dorsal horn and DRG as well as sciatic nerve were dissected to be fused in lysis buffer. The mixture was incubated for 30 minutes at RT and then centrifuged at 13,000 × rpm. After discarding the pellet and collecting the supernatant, the protein content was determined using a BCA Protein Assay Kit. Equal amounts of proteins from each sample were loaded, separated by SDS‐PAGE, and transferred onto a PVDF membrane. The membrane was subsequently blocked for 1 h in 5% nonfat dry milk, washed in Tris‐buffered saline (TBS) for several times, and overnight incubated with primary antibodies at 4°C: anti‐phospho‐Akt antibody (Ser473) (1:500, #9271, Cell Signalling Technology), anti‐Akt antibody (1:500, #9272, Cell Signalling Technology), anti‐PKC‐δ antibody (1:500, 610398, BD), and anti‐β‐actin antibody (1:10000, MAB1501R, Millipore). After washing in in TBS‐ Tween‐20, the membrane was incubated with HRP‐conjugated secondary antibodies for 1 h at RT. At last, an ECL reagent was used to detect the immunoreactive bands. The density of each band was estimated by Sage Creation MiniChemi™ chemiluminescent system and compared to the β‐actin.

### 2.5 Data analyses

All findings in the study were shown as the mean ± standard error of means (SEM). As a multiple comparison analysis, results were scrutinized by using an ANOVA test followed by a Bonferroni test. Otherwise, results were scrutinized by using an unpaired two‐tailed *t* test when comparing two datasets. According to a criterion of *p* < 0.05, differences were set statistically significant.

## 3 RESULTS

### 3.1 CDDO administration ameliorated PHN

Before RTX injection, there was no significant difference in paw withdrawal mechanical threshold. Nociceptive behaviour was assessed at baseline, Days 3 and 7. Compared with the control group, mechanical sensitivity significantly decreased in PHN‐rats (Figure 1B), like our previous study.^11^ TRPV1, a nociceptive sensor, can precept noxious stimulus to regular chronic pain.^21^ To estimate which neuronal type is involved, we obtained the results by dual immunofluorescent images stained with NeuN (a marker for the neuron) and TRPV1 staining. Photographs revealed that the nociceptive neurons were increased in the sciatic nerve (Figure 1C), DRG (Figure 1D) and spinal dorsal horn (Figure 1E) in PHN‐rats, compared to that of control group. CDDO exposure reduced the number of TRPV1‐positive neurons of sciatic nerve, DRG and spinal dorsal horn in PHN‐rats. Therefore, treatment with CDDO significantly ameliorated the mechanical hypersensitivity in PHN‐rats by decreasing TRPV1‐positive nociceptive neurons.

### 3.2 CDDO administration reduced neuronal apoptosis in sciatic nerve, DRG and spinal dorsal horn in PHN‐rats

**FIGURE 2:**
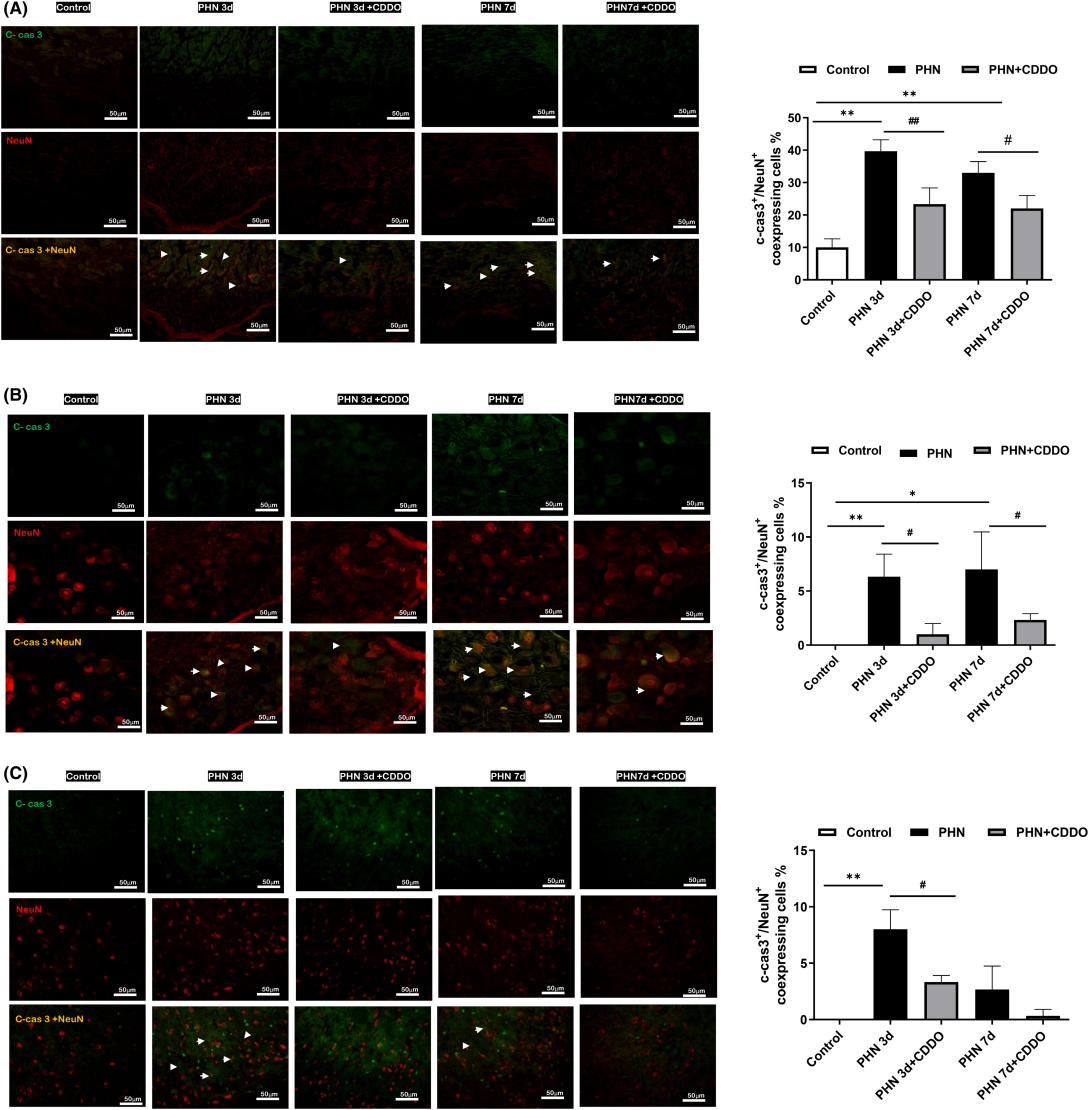
CDDO administration reduced neuronal apoptosis in sciatic nerve, DRG and spinal dorsal horn in PHN‐rats (A) The neuronal cell marker, NeuN (red) and cleaved caspase 3 (C‐cas3) staining, representative photograph in sciatic nerve. Quantification of the percentage of cleaved‐caspase 3‐positive neuronal cells over NeuN‐ positive cells. Scale bars = 50 μm. (B) The neuronal cell marker, NeuN (red) and cleaved caspase 3 (C‐cas3) staining, representative photograph in DRG. Quantification of the percentage of cleaved‐caspase 3‐positive neuronal cells over NeuN‐ positive cells. Scale bars = 50 μm. (C) The neuronal cell marker, NeuN (red) and cleaved caspase 3 (C‐cas3) staining, representative photograph in spinal dorsal horn. Quantification of the percentage of cleaved‐caspase 3 positive neuronal cells over NeuN‐ positive cells. Scale bars = 50 μm. Yellow fluorescence showed colocalization of cleaved‐caspase 3 (green) with NeuN (red). The quantitative data shown as the mean ± SEM. Arrowheads indicated apoptotic neurons. (*n* = 3/group) (*p < 0.05, **p < 0.01, PHN compared with the control group; #*p* < 0.05, ##*p* < 0.01, PHN compared with the indicated PHN + CDDO group).

Following nerve injury, sensory neurons develop cell death in the DRG and spinal dorsal horn.^22^ Since an i.p. capsaicin injection can induce the death of the neurons,^23^ dual immunofluorescent images stained with NeuN and cleaved‐caspase 3 were assessed to determine whether neuronal death is involved in the PHN‐rats. Photographs revealed that the apoptotic neurons stained by cleaved‐caspase3‐positive were increased in the sciatic nerve (Figure 2A), DRG (Figure 2B) and spinal dorsal horn (Figure 2C) in PHN‐rats, compared to that of control group. CDDO exposure reduced the number of cleaved‐caspase3‐positive of sciatic nerve, DRG and spinal dorsal horn in PHN‐rats. These findings above confirmed that CDDO could reduce apoptotic neurons in sciatic nerve, DRG and spinal dorsal horn of PHN‐rats to ameliorate neuropathic pain.

### 3.3 CDDO administration reduced glial cell reaction in sciatic nerve, DRG and spinal dorsal horn in PHN‐rats

**FIGURE 3:**
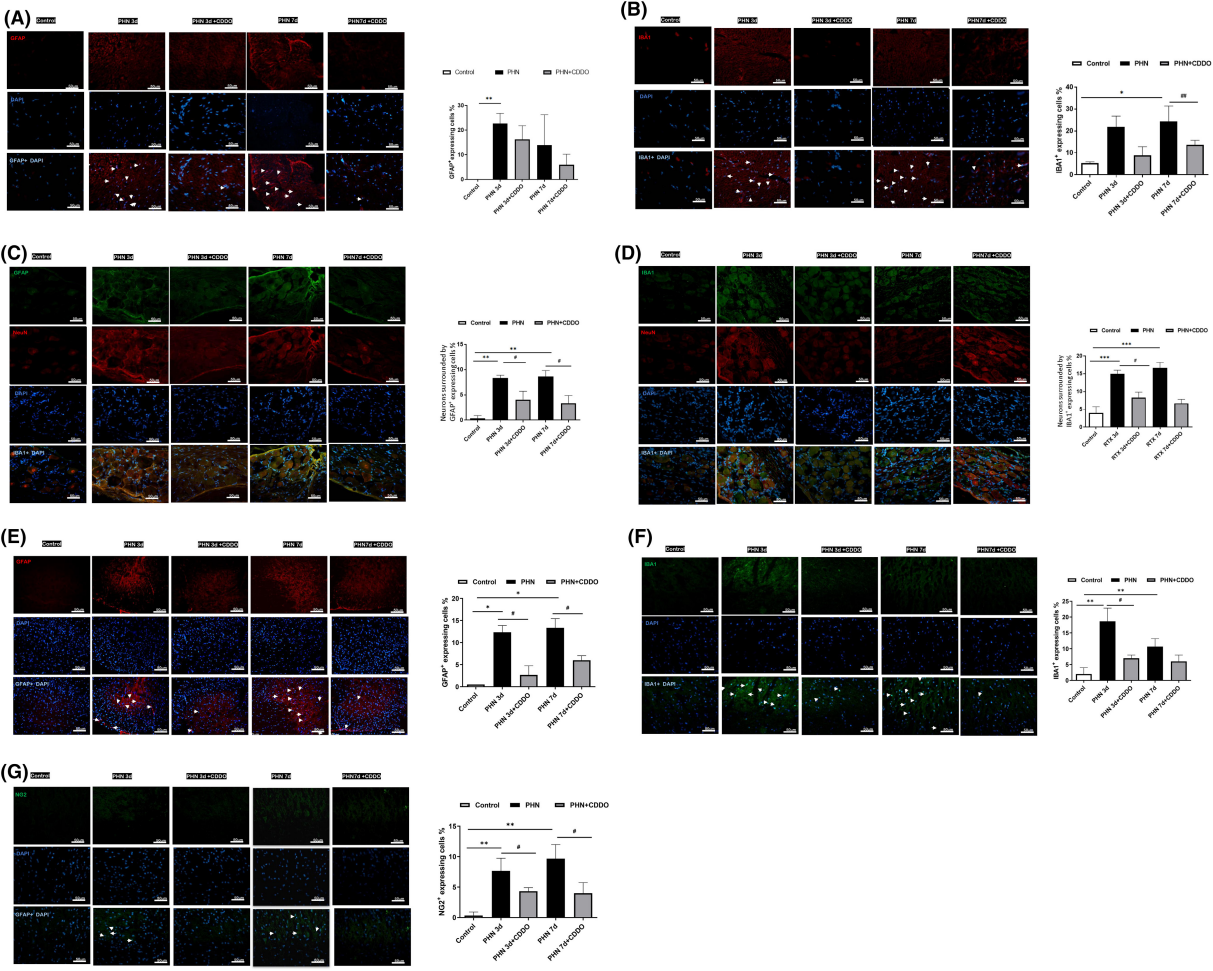
CDDO administration reduced glial cell reaction in sciatic nerve, DRG and spinal dorsal horn in PHN‐rats. (A) The satellite glial cell marker GFAP (red) and DAPI counterstaining, representative photograph. Quantification of the percentage of GFAP‐positive stained cells over DAPI positive cells in sciatic nerve. Merge showed co‐localization of DAPI (blue) with GFAP (red) with indicated arrowheads. (B) The activated macrophage marker IBA1 (red) and DAPI counterstaining, representative photograph. Quantification of the percentage of IBA1‐positive stained cells over DAPI positive cells in sciatic nerve. Merge showed co‐localization of DAPI (blue) with IBA1 (red) with indicated arrowheads. Scale bars = 50 μm. (C) The satellite glial cell marker GFAP (red) and DAPI (blue) counterstaining, representative photograph. Quantification of the percentage of neurons surrounded by GFAP‐positive stained cells over total neurons in DRG. (D) The activated macrophage marker IBA1 (green) and DAPI (blue) counterstaining, representative photograph. Quantification of the percentage of neurons surrounded IBA1‐positive stained cells over total neurons in DRG. Scale bars = 50 μm. (E) The astrocyte marker GFAP (red) and DAPI counterstaining, representative photograph. Quantification of the percentage of GFAP‐positive stained cells over DAPI positive cells. Merge showed co‐localization of DAPI (blue) with GFAP (red) with indicated arrowheads. (F) The microglia marker IBA1 (green) and DAPI counterstaining, representative photograph. Quantification of the percentage of IBA1‐positive stained cells over DAPI positive cells. Merge showed co‐localization of DAPI (blue) with IBA1 (green) with indicated arrowheads. (G) The oligodendrocyte marker NG2 (red) and DAPI counterstaining, representative photograph. Quantification of the percentage of NG2‐positive stained cells over DAPI positive cells. Merge showed co‐localization of DAPI (blue) with NG2 (red) with indicated arrowheads. Scale bars = 50 μm. The quantitative data shown as the mean ± SEM (*n* = 3/group) (*p < 0.05, **p < 0.01, ***p < 0.001, PHN compared with the control group; #*p* < 0.05, ##*p* < 0.01, PHN compared with the indicated PHN + CDDO group).

Studies showed that glial cells rapidly activated in the RTX neuropathy, which is rescued after treatment with a glial inhibitor.^11^, ^24^ To determine whether the activation of glial cells is involved in the PHN‐rats, immunofluorescent images were assessed. Photographs substantiated that the GFAP (a marker for satellite glial cell in sciatic nerve and DRG, and the astrocyte in spinal cord)‐positive cells in the sciatic nerve (Figure 3A), DRG (Figure 3C) and spinal dorsal horn (Figure 3E) were significantly activated, compared to that of control group. CDDO exposure decreased the number of GFAP‐positive cells. IBA1 (a marker for activated macrophage in in sciatic nerve and DRG, and the microglia in spinal cord) positive cells in the sciatic nerve (Figure 3B), DRG (Figure 3D) and spinal dorsal horn (Figure 3F) were significantly activated, compared to that of control group. CDDO exposure decreased the number of IBA1‐positive cells. In addition, NG2 (a marker for oligodendrocytes in spinal cord) positive cells in the spinal dorsal horn was significantly activated, compared to that of control group. CDDO exposure decreased the number of NG2‐positive cells (Figure 3G). These data implies that satellite glial cell and activated macrophages in sciatic nerve and DRG, oligodendrocytes, astrocyte and microglia in spinal dorsal horn proliferated in PHN. i.p. administration of CDDO promisingly inhibited glial cell reaction in PHN‐rats.

### 3.4 CDDO administration attenuated expression of PKC and phosphorylated Akt (p‐Akt) in the sciatic nerve, DRG and spinal dorsal horn in PHN‐rats

**FIGURE 4:**
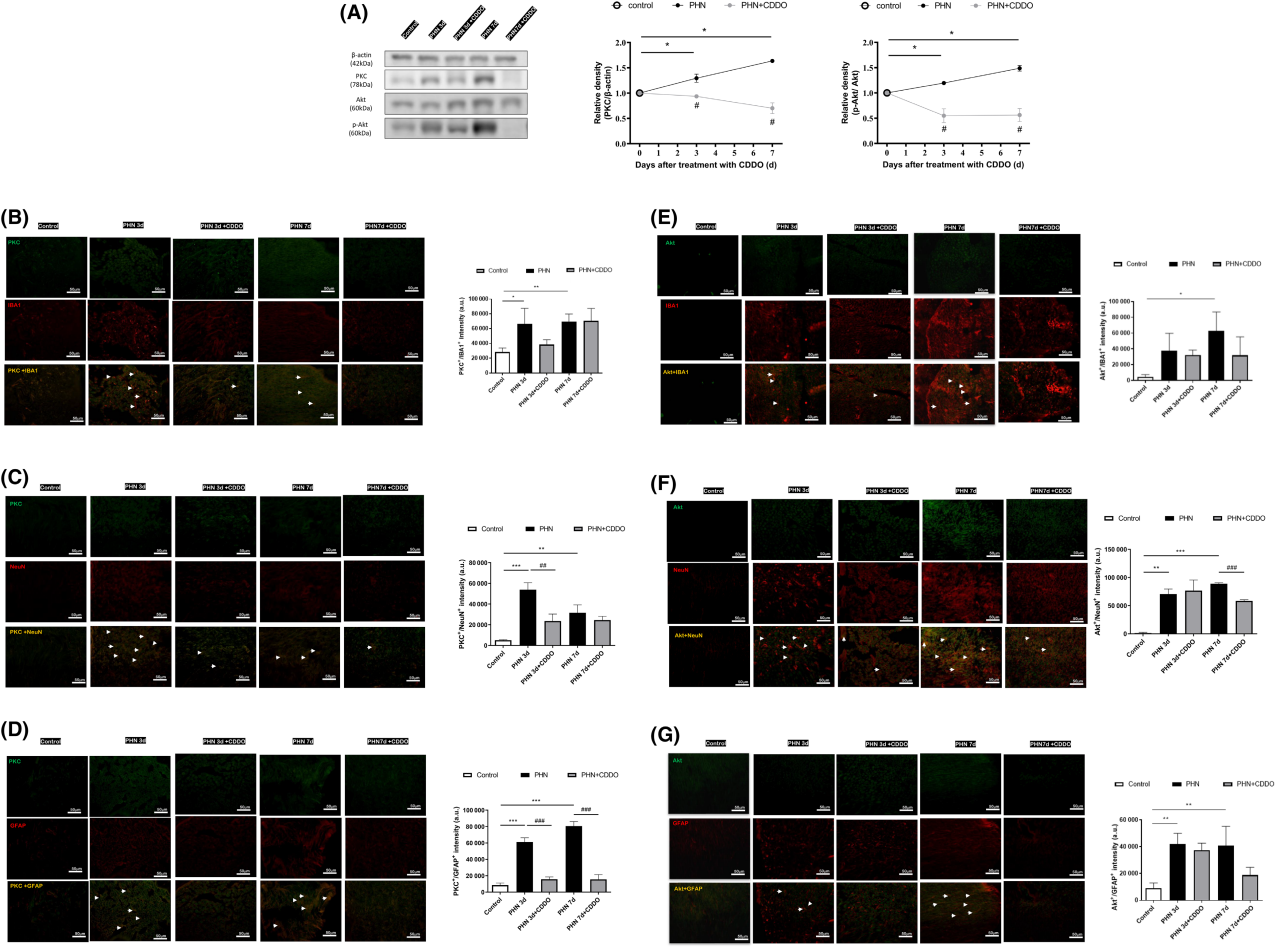
CDDO administration regulated neuron–glia interaction through PKC‐δ and Akt signalling in sciatic nerve of PHN‐rats. (A) Western blot images of PKC‐δ, Akt and *p*‐Akt protein (Ser473) in sciatic nerve. The quantitative blot density (normalized to the β‐Actin loading control) of sciatic nerve. (B) PKC‐δ was expressed in activated macrophage, neurons, and satellite glial‐cell in sciatic nerve. The PKC‐δ positive cells were double stained with antibodies to PKC‐δ (green) and IBA1 (C) NeuN and (D) GFAP (red), Scale bars = 50 μm. Yellow fluorescence showed colocalization of merged cells. Akt was expressed in activated macrophage, neurons, and satellite glial cell in sciatic nerve. The Akt positive cells were double stained with antibodies to Akt (green) and (E) IBA1, (F) NeuN, and (G) GFAP (red), Scale bars = 50 μm. Yellow fluorescence showed colocalization of merged cells. The quantitative data shown as the mean ± SEM (*n* = 3/group) (*p < 0.05, **p < 0.01, ***p < 0.001, PHN compared with the control group; #*p* < 0.05, ##*p* < 0.01, ###*p* < 0.001, PHN compared with the indicated PHN + CDDO group).

**FIGURE 5:**
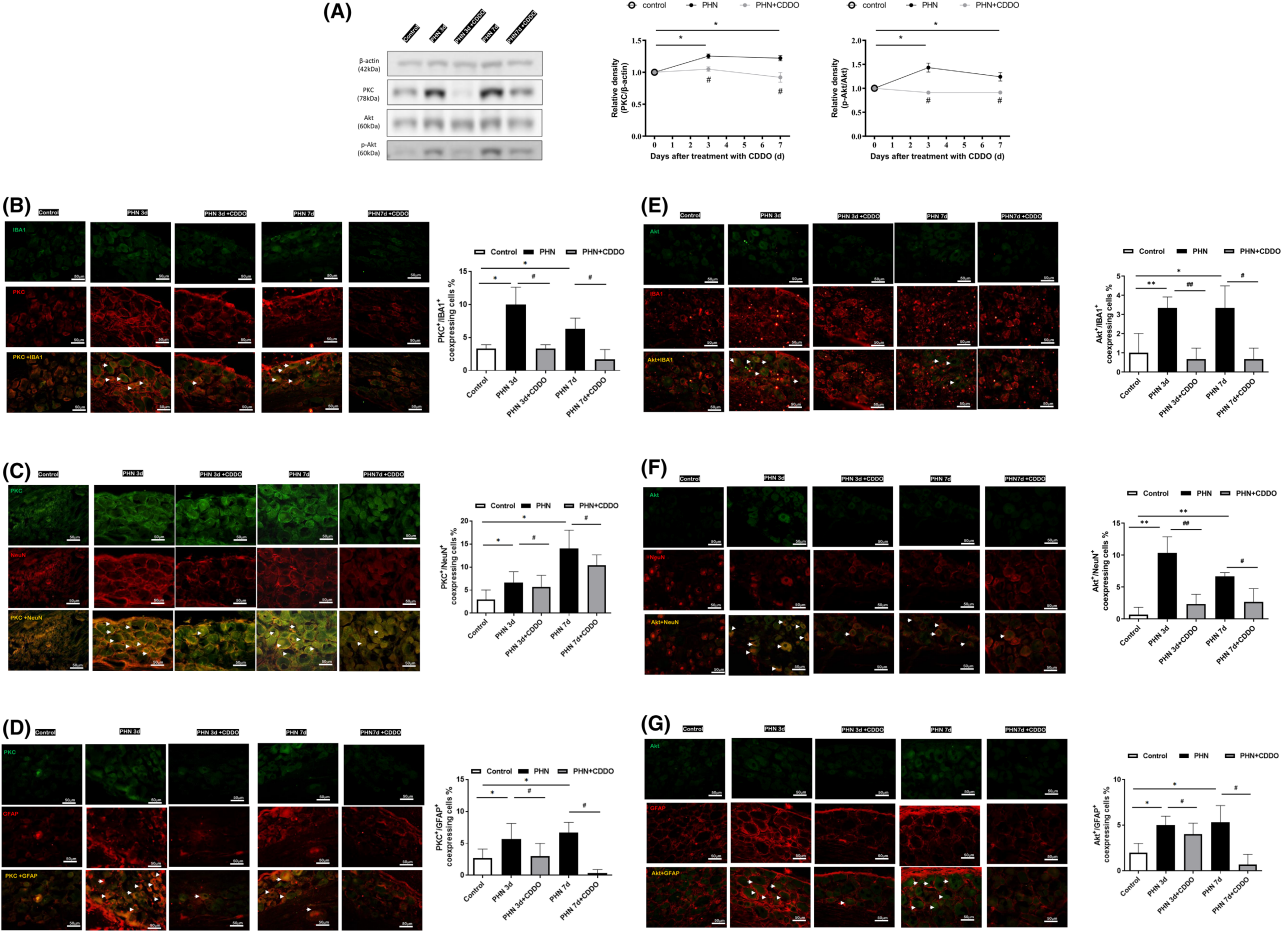
CDDO administration regulated neuron–glia interaction through PKC‐δ and Akt signalling in DRG of PHN‐rats. (A) Western blot images of PKC‐δ, Akt and p‐Akt protein (Ser473) in DRG. The quantitative blot density (normalized to the β‐Actin loading control) of DRG. PKC‐δ was expressed in activated macrophage, neurons, and satellite glial‐cell in DRG. (B) The PKC‐δ positive cells were double stained with antibodies to PKC‐δ (red) and IBA1(green), Scale bars = 50 μm, (C) PKC‐δ (green) and NeuN (red), Scale bars = 50 μm, and (D) GFAP (red), Scale bars = 50 μm. Yellow fluorescence showed colocalization of merged cells. Akt was expressed in activated macrophage, neurons, and satellite glial cell in DRG. The Akt positive cells were double stained with antibodies to Akt (green) and (E) IBA1 (red), Scale bars = 50 μm, (F) NeuN (red), Scale bars = 50 μm, and (G) GFAP (red), Scale bars = 50 μm. Yellow fluorescence showed colocalization of merged cells. The quantitative data shown as the mean ± SEM (*n* = 3/group) (*p < 0.05, **p < 0.01, PHN compared with the control group; #*p* < 0.05, ##*p* < 0.01, PHN compared with the indicated PHN + CDDO group).

**FIGURE 6:**
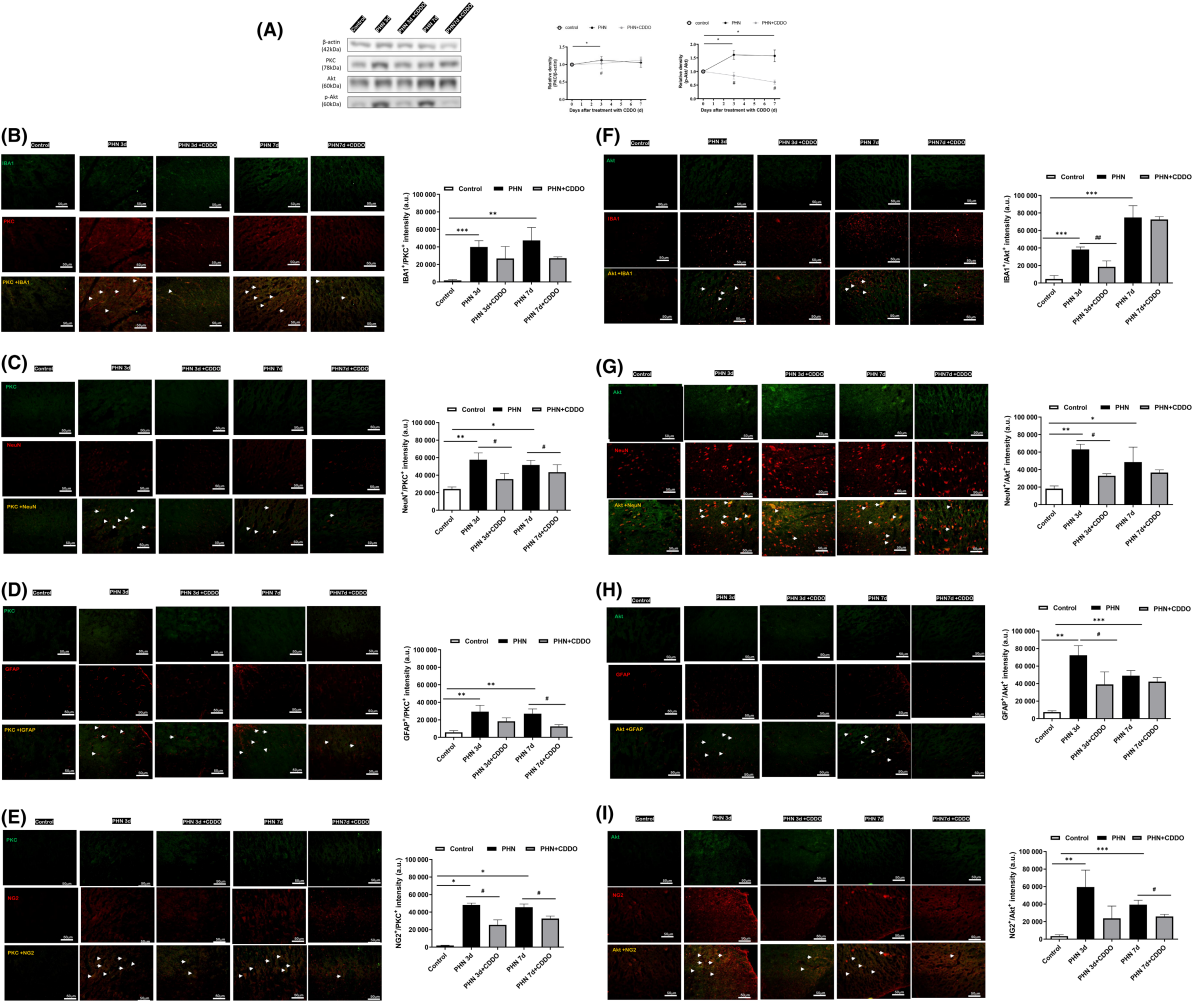
CDDO administration regulated neuron–glia interaction through PKC‐δ and Akt signalling in spinal dorsal horn of PHN‐rats. (A) Western blot images of PKC‐δ, Akt and p‐Akt protein (Ser473) in spinal dorsal horn. The quantitative blot density (normalized to the β‐Actin loading control) of spinal dorsal horn (B) PKC‐δ was expressed in microglia, neurons, astrocyte, and oligodendrocyte in spinal dorsal horn. The PKC‐δ positive cells were double stained with antibodies to PKC‐δ (green) and IBA1, (C) NeuN (D) GFAP, and (E) NG2 (red), Scale bars = 50 μm. Yellow fluorescence showed colocalization of merged cells. Akt was expressed in microglia, neurons, astrocyte, and oligodendrocyte in spinal dorsal horn. The Akt positive cells were double stained with antibodies to Akt (green) and (F) IBA1, (G) NeuN (H) GFAP, and (I) NG2 (red), Scale bars = 50 μm. Yellow fluorescence showed colocalization of merged cells. The quantitative data shown as the mean ± SEM (*n* = 3/group) (*p < 0.05, **p < 0.01, ***p < 0.001, PHN compared with the control group; #*p* < 0.05, ##*p* < 0.01, PHN compared with the indicated PHN + CDDO group).

Protein kinase C (PKC) signalling is known to participate in the central or peripheral sensitization in nociceptive transmission. Application of capsaicin on skin can generate the formation of PKC to produce hyperalgesia.^25^ PKC‐δ in DRG can mediate spontaneous pain in peripheral neuropathy.^26^ In modulating nociceptive information, p‐Akt is widely expressed in spinal dorsal horn and DRG. Plantar excision can induce the activation of Akt in spinal neurons and microglia in mice model.^27^ Intradermal capsaicin administration also cause Akt activation in the lumbar spinal cord.^28^ Since the PKC‐δ and Akt signalling are also important in regulating neuronal apoptosis, western blot was arranged to determine whether PKC‐δ or Akt signalling are activated in PHN‐rats. Systemic RTX significantly rose the expressions of PKC‐δ and phosphorylated Akt (Ser473) in the sciatic nerve (Figure 4A), DRG (Figure 5A), and spinal dorsal horn (Figure 6A), compared to that of control group. CDDO exposure significantly decreased the expressions of PKC‐δ and phosphorylated Akt (Ser473), in accordance with the behaviour results. These results suggest that CDDO can prevent activation of PKC‐δ and Akt signalling pathways in PHN‐rats.

### 3.5 CDDO administration regulated neuron–glia interaction through PKC‐δ and Akt signalling in PHN‐rats

To characterize the cell type that expressed PKC‐δ and Akt in sciatic nerve, DRG and spinal dorsal horn in PHN‐rats, the distribution of PKC‐δ and Akt was labelled by double immunofluorescence staining (IBA1, NeuN, GFAP, and/or NG2). Both PKC‐δ and Akt were co‐localized with IBA1, NeuN, and GFAP in sciatic nerve (Figure 4B–G) and DRG (Figure 5B–G). Both PKC‐δ and Akt were co‐localized with IBA1, NeuN, GFAP, and NG2 in spinal dorsal horn (Figure 6B–I). Hence, PKC‐δ and Akt were expressed in activated macrophages, neurons, and satellite glial cells in sciatic nerve and DRG. PKC‐δ and Akt were expressed in microglia, neurons, astrocytes, and oligodendrocytes in spinal dorsal horn. The results suggested that RTX induced neuron–glia interaction during central and peripheral sensitization through PKC‐δ and Akt signalling, causing neuronal apoptosis.

## 4 DISCUSSION

**FIGURE 7:**
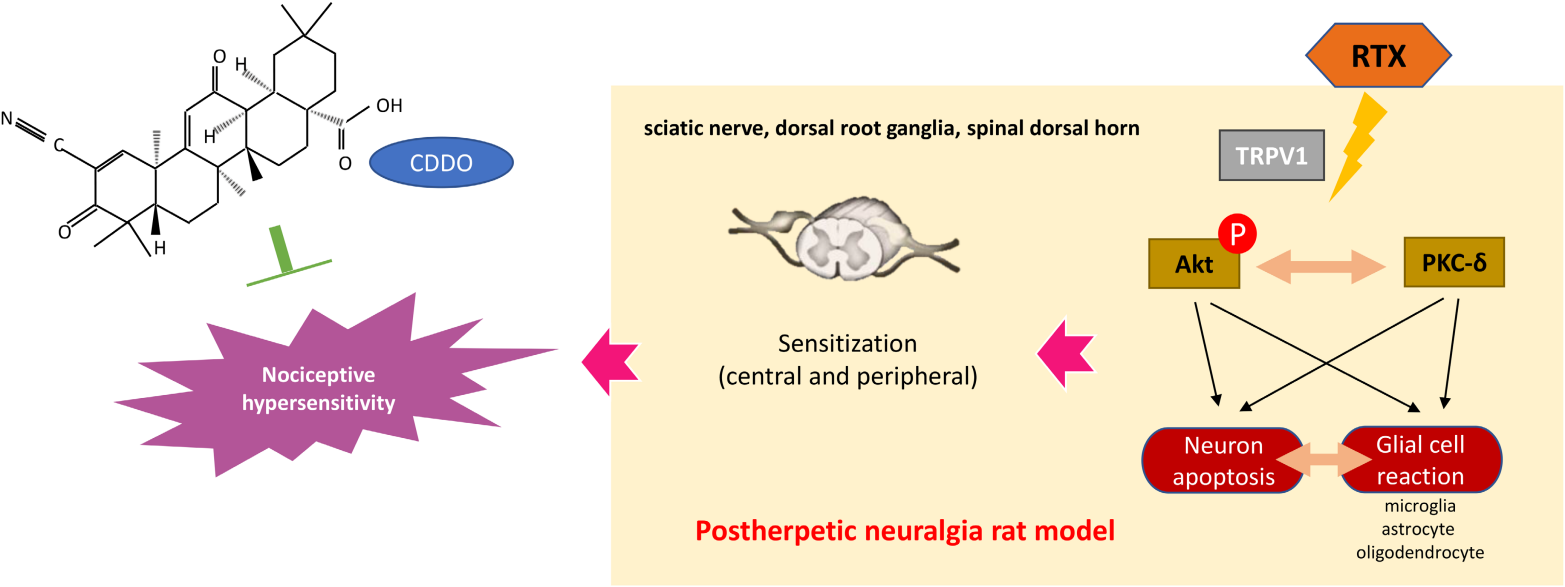
Analgesic action of CDDO by disrupting PKC‐δ and phosphorylated Akt (Ser473) in PHN‐rats. After systemic treatment with RTX, glial cell reaction causes neuronal apoptosis in sciatic nerve, DRG and spinal dorsal horn, which leads to PHN. However, CDDO reduces nociceptive hypersensitivity, rescues apoptotic neurons, and restores glial reaction by reversing PKC‐δ and p‐Akt (Ser473) in PHN‐rats.

This is a promising report documenting the therapeutic action of CDDO in a PHN‐rat's model and it has potential to treat neuropathic pain. In the present study, behavioural testing indicated that systemic RTX substantially induced persistent profound mechanical hyperalgesia in adult PHN‐rats, like our previous report.^11^ CDDO significantly alleviated the mechanical hyperalgesia in PHN‐rats. Consistently, CDDO reduced the apoptotic neurons in sciatic nerve, DRG and spinal dorsal horn. CDDO also reversed the glial cell reaction in sciatic nerve, DRG and spinal dorsal horn. Furthermore, CDDO inhibited the expressions of PKC‐δ and phosphorylated Akt (Ser473) in the sciatic nerve, DRG and spinal dorsal horn. Therefore, our study implied that CDDO may improve mechanical sensitivities and exert a neuroprotective effect on a PHN‐rat's model by ameliorating RTX‐induced damage to nerve, activation of microglia (activated macrophage), astrocytes (satellite glial cell) or oligodendrocytes, as well as suppression of PKC‐δ and phosphorylated Akt (Ser473) in sciatic nerve, DRG and spinal dorsal horn (Figure 7).

Injury to nerve and sensitization of nociceptors lead to neuropathic pain, causing profound disabilities.^29^ The transmission of pain signals starts from peripheral sensory neurons through the spinal dorsal horn to the supraspinal structures, where the spinal dorsal horn is the first relay center. Sensory neuronal cell death occurs in the DRG and spinal dorsal horn following nerve injury.^22^ Leukocytes infiltrate to damaged nerve and activate the resident macrophages and Schwann cells. NF‐κB pathway is further activated to upregulate the expression of iNOS with coordination of TNFα.^30^ Through the activation of downstream caspases, extrinsic and intrinsic pathways induce the neuronal apoptosis.^31^ Capsaicin can produce toxic effects on cultures of rat sensory neurons, generate nNOS to damage neurons innervating the stomach in adult rats and induce neuronal death in DRG by i.p. treatment.^32^ Previous studies have proved that RTX depletes unmyelinated afferent neurons and injuries myelinated afferent fibres.^33^ By increasing TNFα, RTX leads to the release of pro‐apoptotic factors to cause neuronal cell apoptosis to induce neuropathic pain.^34^ In this study, the number of apoptotic neurons in the sciatic nerve, DRG and spinal dorsal horn were increased in PHN‐rats, which was rescued by CDDO. Therefore, the present study indicates that the CDDO could inhibit neuronal apoptosis in the sciatic nerve, DRG and spinal dorsal horn to attenuate mechanical hyperalgesia in rats.

Activation of glial cells throughout spinal cord, cortex as well as peripheral nerve, and hyperactivation of proinflammatory responses mediates the development of neuropathic pain.^35^ Glial cells, microglia, astrocytes, and oligodendrocytes undergo structural and functional modifications.^36^, ^37^ At the early phase of neuropathic pain, microglia are activated to become proliferation and hypertrophy, and accompanied by upregulation of immune surface antigens and phosphorylation of MAP kinases.^38^ Also, the p38 activation in microgliosis turns on NF‐κB, which causes the release of IL‐1 or TNFα to induce the transformation of astrocytes.^39^ Astrocytes are further activated and synapse during the sustainment phase. Following nerve injury, astrocytes may proliferate, become hypertrophy, and reactive to release signalling molecules to cause pain.^40^ Hence, they secrete neurotoxins to cause rapid death of neurons and oligodendrocytes due to neuroinflammation.^41^ Besides, both astrocytes and microglia activate oligodendrocytes to enhance remyelination.^42^ Oligodendrocytes release IL‐33 to activate ST2 receptors on spinal microglia to regulate neuropathic pain.^43^ By astrocyte‐microglia crosstalk, astrocytes can regulate microglial phenotypes and phagocytosis. By astrocyte‐neuron interactions, astrocytes also control excitatory synaptic transmission.^44^ Through oligodendrocyte‐microglia‐astrocyte crosstalk, glial cells cooperate to initiate and maintain the neuropathic pain.^45^ Previous studies have revealed that RTX induces the activation of astrocytes and microglia, whereas glial inhibitors reverse the nociceptive hyperalgesia.^24^ Our previous study also demonstrated blocking HDGF attenuated mechanical hyperalgesia by inhibiting astrocyte reaction in the spinal cord.^11^ In this study, GFAP‐positive satellite glial cells, IBA1‐postive activated macrophages in sciatic nerve and DRG, GFAP‐positive astrocytes, IBA1‐positive microglia, NG2‐positve oligodendrocytes in spinal dorsal horn were increased in PHN‐rats, whereas CDDO further inhibited the reaction. The present study demonstrated that the CDDO could attenuate the glial cell reaction in the sciatic nerve, DRG and spinal dorsal horn to improve the mechanical hyperalgesia in PHN‐rats.

Akt signalling is crucial in the formation and maintenance of pain to regulate nociceptive information, which phosphorylated Akt (p‐Akt) is the active conformation.^28^ It is not only expressed in DRG but also in the laminae I‐IV of the spinal dorsal horn, where nociceptive primary afferent fibres terminate.^46^ In chronic post‐surgical pain mice model, the activation of Akt in spinal neurons and microglia can be induced by plantar skin/muscle incision and retraction. Also, microglia can generate the transformation of reactive astrocytes through regulation of Akt.^27^ Studies showed that astrocytic conversion can be modulated in Aβ42‐activated microglia‐conditioned medium via Akt pathway.^47^ Intradermal capsaicin injection leads to the activation of Akt in spinal dorsal horn neurons. Pre‐treatment of Akt inhibitors blocks the mechanical hypersensitivity caused by intradermal capsaicin injection.^28^ Moreover, the Akt phosphorylation signalling contributes to both neuronal cell apoptosis and proliferation. It not only blocks Bad and caspase 9, inhibits glycogen synthase kinase‐3b, but also induces NF‐κB dependent anti‐apoptotic genes to increase neuronal resistance to apoptosis.^48^ On the contrary, studies indicate that Akt serves as a death kinase to regulate programmed cell death in neuronal cells. By mediating downstream mTOR substrate, Akt may promote cell death in hippocampal neuronal cell line HT22.^49^ Both activation of Akt signalling and nuclear translocation of Akt lead to cell death^50^ whereas Akt inhibitors could prevent cultured neurons from photodynamic induced cell death.^51^ Our previous study also demonstrated that blocking HDGF decreases mechanical hyperalgesia by inhibiting Akt pathway in the spinal cord.^11^ In sciatic nerve and DRG, Akt was expressed in satellite glial cell, activated macrophage, and neurons. In spinal dorsal horn, Akt was expressed in oligodendrocytes, astrocytes, microglia, and neurons. In this study, the expression of p‐Akt (Ser473) was increased in the sciatic nerve, DRG and spinal dorsal horn in PHN‐rats, which was reversed by CDDO. The present study indicated that the analgesic effect of CDDO might be mediated through p‐Akt product‐induced activities.

PKC serves as a major effector to modulate neuronal signalling in pain processing since it is distributed from brain to peripheral site of injury.^25^ The activation of PKC can depolarize and sensitize afferent neurons to enhance noxious stimulus, whereas PKC inhibitors could block the pain sensitization. Through activation of the TRPV1 receptor, PKC can modulate nociception transmission.^52^ Application of capsaicin to the skin can lead to thermal hyperalgesia by increasing the expression and depolarization of TRPV1 or the release of inflammatory mediators in a PKC‐dependent manner.^53^ Also, PKC can increase excitatory tone and reduce the inhibitory transmission at synaptic terminal by the release of neuropeptides and excitatory amino acids. In diabetic neuropathy, PKC activation underlies the neuronal sensitization to produce hyperalgesia.^54^ PKC‐δ mediates chronic headache in a nitroglycerin mice model. In paclitaxel‐induced peripheral neuropathy, PKC‐δ evokes pain and mediates spontaneous pain. Series studies showed that the activation of PKC‐δ participates in cell proliferation and death.^55^ PKC‐δ might regulate neuronal cell survival though NF‐κB, Akt, and ERK signalling.^56^ The release of glutamate and accumulation of reactive oxygen species cause the activation of PKC‐δ in ischemic stroke. PKC‐δ can release cytochrome c from the mitochondria through the BAD pathway to induce the apoptotic pathway.^57^ In cancer and immune cells, PKC‐δ mediates PI3‐kinase‐ dependent activation of Akt (Thr308) to regulate cell survival.^58^ In diabetic rats, PKC‐δ modulate retinal neuronal cell apoptosis by regulating downstream Akt signals (Ser473) independent of PI3‐kinase.^59^ Previous study showed that RTX induces the activation of microglia to generate PKC‐δ, thus mediating signal transduction of hyperalgesia.^60^ In sciatic nerve and DRG, Akt was expressed in satellite glial cell, activated macrophage, and neurons. In spinal dorsal horn, Akt was expressed in oligodendrocytes, astrocytes, microglia, and neurons. In PHN‐rats, the expression of PKC‐δ and p‐Akt (Ser473) was increased in the sciatic nerve, DRG and spinal dorsal horn, which was reduced by CDDO and consistent with the glial cell reaction and apoptotic neuronal activities. The present study indicated that the analgesic action of CDDO might be mediated through inhibition of PKC‐δ and p‐Akt (Ser473) signalling.

## 5 CONCLUSIONS

Taken together, this study has revealed a novel direction to treat PHN. CDDO administration improved neuropathic pain, alleviating mechanical hypersensitivity by decreasing TRPV1‐nociceptive neurons in PHN‐rats. The study implies that CDDO exerts the analgesic action which is potentially mediated by glial cells reaction, apoptotic neurons, and the involved PKC‐δ and phosphorated Akt singling. Therefore, CDDO is a potential promising therapeutic analgesic. However, further study is warranted to explore if CDDO still remains beneficial in a virus‐based PHN model.

## AUTHOR CONTRIBUTIONS

**Chun‐Ching Lu:** Conceptualization (equal); formal analysis (equal); investigation (equal); writing – original draft (lead). **Chia‐Yang Lin:** Data curation (equal); formal analysis (equal). **Ying‐Yi Lu:** Funding acquisition (equal); project administration (equal). **Hung‐Pei Tsai:** Investigation (equal); methodology (equal). **Chih‐Lung Lin:** Formal analysis (equal). **Chieh‐Hsin Wu:** Conceptualization (equal); funding acquisition (equal); project administration (equal); supervision (lead); validation (lead); writing – review and editing (lead).

## CONFLICT OF INTEREST STATEMENT

The authors confirm that there are no conflicts of interest.

## Data Availability

All data generated or analysed during this study are included in this published article.
